# Prevalence and risk of progression of preclinical Alzheimer’s disease stages: a systematic review and meta-analysis

**DOI:** 10.1186/s13195-018-0459-7

**Published:** 2019-01-15

**Authors:** Lucilla Parnetti, Elena Chipi, Nicola Salvadori, Katia D’Andrea, Paolo Eusebi

**Affiliations:** Centre for Memory Disturbances, Lab of Clinical Neurochemistry, Section of Neurology, Department of Medicine, University of Perugia, Perugia, Italy

**Keywords:** Preclinical Alzheimer’s disease, Systematic review, Biomarkers, National Institute on Aging–Alzheimer’s Association criteria, Prevalence

## Abstract

**Background:**

Alzheimer’s disease (AD) pathology begins several years before the clinical onset. The long preclinical phase is composed of three stages according to the 2011National Institute on Aging and Alzheimer’s Association (NIA-AA) criteria, followed by mild cognitive impairment (MCI), a featured clinical entity defined as “due to AD”, or “prodromal AD”, when pathophysiological biomarkers (i.e., cerebrospinal fluid or positron emission tomography with amyloid tracer) are positive. In the clinical setting, there is a clear need to detect the earliest symptoms not yet fulfilling MCI criteria, in order to proceed to biomarker assessment for diagnostic definition, thus offering treatment with disease-modifying drugs to patients as early as possible. According to the available evidence, we thus estimated the prevalence and risk of progression at each preclinical AD stage, with special interest in Stage 3.

**Methods:**

Cross-sectional and longitudinal studies published from April 2008 to May 2018 were obtained through MEDLINE-PubMed, screened, and systematically reviewed by four independent reviewers. Data from included studies were meta-analyzed using random-effects models. Heterogeneity was assessed by *I*^2^ statistics.

**Results:**

Estimated overall prevalence of preclinical AD was 22% (95% CI = 18–26%). Rate of biomarker positivity overlapped in cognitively normal individuals and people with subjective cognitive decline. The risk of progression increases across preclinical AD stages, with individuals classified as NIA-AA Stage 3 showing the highest risk (73%, 95% CI = 40–92%) compared to those in Stage 2 (38%, 95% CI = 21–59%) and Stage 1 (20%, 95% CI = 10–34%).

**Conclusion:**

Available data consistently show that risk of progression increases across the preclinical AD stages, where Stage 3 shows a risk of progression comparable to MCI due to AD. Accordingly, an effort should be made to also operationalize the diagnostic work-up in subjects with subtle cognitive deficits not yet fulfilling MCI criteria. The possibility to define, in the clinical routine, a patient as “pre-MCI due to AD” could offer these subjects the opportunity to use disease-modifying drugs at best.

**Electronic supplementary material:**

The online version of this article (10.1186/s13195-018-0459-7) contains supplementary material, which is available to authorized users.

## Background

Alzheimer’s disease (AD) is a progressive disorder with a long-lasting asymptomatic phase followed by a symptomatic predementia phase, and finally by the dementia stage [1, 2].

Based on the dynamical model driven by the amyloid cascade, the pathophysiological AD processes begin several years before the onset of clinical symptoms [1]. These pathophysiological processes taking place in the brain are reliably detectable in vivo by cerebrospinal fluid (CSF) or imaging biomarkers, which are needed for diagnosing AD in the predementia phase [1, 3–7]. In recent years, the linear model in which a mechanical sequence occurs—starting from amyloidosis and then leading to neuronal injury—has been questioned, since cases of neurodegeneration preceding incipient brain amyloid pathology have been described [8–12]. The research framework for preclinical AD [13, 14] recently proposed a descriptive system, based on a categorical classification of biomarker positivity (A/T/N): “A” refers to amyloid pathology, assessed by either Aβ42 or the Aβ42/Aβ40 ratio in CSF or amyloid PET; “T” refers to tau pathology, assessed by CSF phospho-tau or PET with tau tracer; and “N” refers to neurodegeneration, assessed by CSF total tau, MRI, or FDG-PET. This classification model represents an advancement from the 2011 NIA-AA [2, 6] and IWG-2 [4, 5] criteria, since it takes into account several pathophysiological profiles underlying different conditions. All of them may lead to different trajectories, identifying specific prognostic features and cognitive outcomes. In this model, the only presence of brain amyloidosis identifies “AD pathologic change” [14]. Conversely, an early diagnosis of Alzheimer’s disease requires the presence of both amyloidosis (A^+^) and tauopathy (T^+^), where the positivity of an injury biomarker (N) is not mandatory for diagnostic purposes since it represents an unspecific measure of neuronal damage entity. Currently, an attempt has been proposed to compare the NIA-AA preclinical AD stages [2] and the A/T/N classification in cognitively normal individuals proposed in the 2018 NIA-AA Research Framework [15]. Accordingly, NIA-AA Stage 3 seems to correspond to Stage 2 in the 2018 NIA-AA Research Framework.

Pathophysiological AD biomarker positivity represents a mandatory criterion for inclusion in clinical trials with novel therapeutic drugs [4]. Up to now, in clinical terms, the only well-defined symptomatic predementia stage in routine diagnostic work-up is represented by mild cognitive impairment (MCI), a clinical entity in which cognitive deficits do not fulfill dementia criteria [6, 16, 17]. In the last years, an increasing number of papers have been focused on the phase preceding MCI (i.e., “pre-MCI phase”) [18, 19]. Although no shared definition is available, several attempts have been carried out to characterize the symptomatic debut of AD.

In the Alzheimer’s Disease Neuroimaging Initiatives (ADNI, ADNI GO, and ADNI 2), the MCI stage was divided into early MCI (EMCI) and late MCI (LMCI) [20–22]. Both of these are characterized by evidence of AD biomarker abnormalities, where EMCI patients show milder cognitive deficits. In terms of neuropsychological criteria, EMCI is defined as a performance 1–1.5 SD below the mean in one episodic memory test, identifying subtle memory impairment at an intermediate level between normal cognition and MCI [20, 23]. However, this definition remains controversial.

Based on the 2011 National Institute on Aging and Alzheimer’s Association (NIA-AA) staging of preclinical AD [2], cognitive decline emerges in the last step of the preclinical continuum (i.e., Stage 3) as “*subtle cognitive decline*”. However, how this decline could be quantified is not well defined, and a consensus on cognitive measures reliably depicting such subtle deficits is not yet available. Epelbaum et al. [24] suggest an entity named “*Preclinical AD with subtle cognitive changes*” (attention impairment or dysexecutive symptoms) displaying performances less than 1 SD below the age-corrected normative mean in one or more cognitive measure. The concept of what represents a “subtle” decline is challenging also due to recent evidence that subtle signs of cognitive dysfunction may precede Aβ-positivity several years before the threshold for pathological Aβ accumulation is reached [25].

A more extensive neuropsychological evaluation can help in detecting cognitive alterations in pre-MCI phases of AD [2, 11, 26, 27]. Among neuropsychological measures, composite scores (obtained by normalizing and summing up standardized *z* scores) have proved to have higher sensitivity than single scores at cognitive tests in detecting changes over time within the preclinical AD stages [27–33]. Accordingly, they have also been introduced as outcome measures in prevention trials [5, 31, 32, 34].

A feature that can accompany the symptomatic debut of AD is the presence of subjective cognitive concerns. Self-reported experiences of cognitive decline have been defined using different labels—subjective cognitive impairment (SCI) [35], subjective cognitive complaints (SCC) [36], subjective memory impairment (SMI) [37], and subjective memory complaints (SMC) [38]—leading to a highly heterogeneous lexicon. In 2014, the Subjective Cognitive Decline Initiative (SCD-I) working group tried to reduce this complexity by reaching a consensus on terminology, using the definition of subjective cognitive decline (SCD), recently operationalized [39]. SCD refers to a self-experienced decline in some cognitive abilities in comparison with a previous normal status, in spite of normal performance on standardized cognitive tests.

Whether this condition may or may not represent an increased risk for progression to MCI and/or dementia is still a controversial issue [40]. However, a consistent body of evidence indicates that SCD actually represents a risk factor for future cognitive decline, for MCI and AD dementia [30, 40–45], particularly when worries are reported [46–48]. Dubois et al. [5, 49] specify that the SCD per se cannot be considered as a “proxy” for preclinical AD, in line with studies reporting that the percentage of amyloid PET positivity is independent from rates of memory complaints [50, 51]. However, SCD individuals with evidence of pathophysiological AD biomarker positivity (SCD plus) represent a category at risk for clinical AD [39].

According to available papers published between 2008 and 2018, our systematic review aimed to estimate the prevalence of AD pathophysiological biomarker positivity (CSF, amyloid PET) across all individuals lying in the spectrum preceding MCI, including subjects defined as cognitively normal (CN) or in subjective cognitive decline. In these categories, we then calculated the risk of clinical progression to MCI and/or dementia.

## Methods

Our systematic review and meta-analysis was conducted according to the PRISMA guidelines (see Additional file 1: Table S1) [52].

### Data sources and search strategy

We searched MEDLINE (via PubMed) from April 2008 to May 2018. We examined reference lists of all eligible studies and reviews in the field for further possible titles; the process was repeated until no new titles were found.

For the search strategy we used the following terms: “preclinical” OR “asymptomatic” OR “pre-MCI” OR “preMCI” OR “pre MCI” OR “cognitively normal” OR “normal aging” OR “subjective memory complaint*” OR “subjective memory impairment” OR “subjective cognitive complaint*” OR “subjective cognitive impairment” OR “subjective cognitive decline” OR “memory complaint*” OR “cognitive complaint*” OR “subjective cognitive” OR “subjective memory” OR “SCD” OR “subtle cognitive decline” OR “early diagnosis” OR “Stage” AND “Alzheimer*” AND “biomarker” OR “abeta” OR “amyloid-beta” OR “amyloid” OR “tau” OR “t-tau” OR “p-tau” OR “total tau” OR “phospho-tau” OR “phosphorylated tau” OR “hyperphosphorylated tau” OR “PET” OR “positron emission tomography” OR “CSF” OR “cerebrospinal fluid” OR “amyloid PET”.

### Data extraction

Four investigators (EC, NS, PE, and KDA) conducted literature searches and extracted data ensuring two independent evaluations for each record. Disagreements were resolved by means of consensus.

### Inclusion criteria

We limited the search to the previous 10 years, referring to articles following the publication of the IWG criteria and revision of the NINCS-ADRDA by Dubois et al. [53] where the assessment of in-vivo AD biomarkers was considered a fundamental step, supportive to the clinical phenotype, in improving the accuracy of early AD diagnosis. Studies were included if pathophysiological biomarkers (CSF amyloid-β1–42, t-tau, and p-tau, or Aβ42/tau ratio, or amyloid PET including any amyloid tracer and assessment via visual scales or quantitative measures) for defining preclinical AD were used and if CSF biomarkers and amyloid PET resulted negative or positive according to study-specific cutoff points. We have considered prospective and retrospective cohort studies that included one of the following three categories of pre-MCI individuals: cognitively normal (CN), as defined by normal scores on cognitive tests; subjective cognitive decline, as defined by the presence of cognitive complaints associated with normal performance in cognitive tests (we included studies that classified subjects as subjective cognitive decline (SCD), subjective memory complaints (SMC), subjective memory impairment (SMI), subjective cognitive complaints (SCC), and subjective cognitive impairment (SCI), and included people with reported SCD at baseline assessed with any kind of method (e.g., single dichotomous questions, questionnaires, interviews)); and Stage 3 of preclinical AD continuum, as defined in the 2011 NIA-AA criteria [2] by presence of amyloidosis, neurodegeneration, plus subtle cognitive decline (any cognitive decline or impairment in any neuropsychological assessment which does not reach MCI criteria). Studies with fewer than 50 subjects in our groups of interest were excluded. A sample size of 50 was chosen in order to increase the probability of including studies at low/moderate risk of bias. When more than one study reported results of a specific cohort (i.e., ADNI cohort), we included the one with the largest sample size.

### Operationalization criteria for evaluating the risk of progression

In studies reporting longitudinal follow-up we have considered as clinical progression the onset of MCI, progression from normal cognition to MCI or dementia, change in CDR score, cognitive decline in neuropsychological tests, or any of these conditions.

### Risk of bias assessment

The methodological quality of the included studies was evaluated with the tool developed by Hoy et al. [54]. A score of 1 (yes) or 0 (no) was assigned for each item, and scores were summed across items to generate an overall quality score that ranged from 0 to 10. Studies were then classified as having a low (> 8), moderate [6–8], or high (≤ 5) risk of bias. Two investigators independently assessed the study’s methodological quality (EC, NS), with disagreements resolved by a third investigator (PE).

### Data analysis

Data analysis was performed using R software version 3.4, and the meta package was used for meta-analysis [55]. For each study we summarized several characteristics of the participants such as gender, age, years of education, and ethnicity. Prevalence and relative risks (RR) were meta-analyzed using random-effects models. Summary estimates were provided along with 95% confidence intervals (95% CIs). Heterogeneity among studies was assessed using *I*^2^ statistics. When considering the prevalence of preclinical AD, we explored several study-level characteristics as sources of heterogeneity by means of meta-regression models and subgroup analyses. In the meta-analysis of relative risk of progression, publication bias was assessed by means of funnel plot. *P* < 0.05 was considered significant in all of the analyses.

## Results

### Characteristics of included studies

**Fig. 1:**
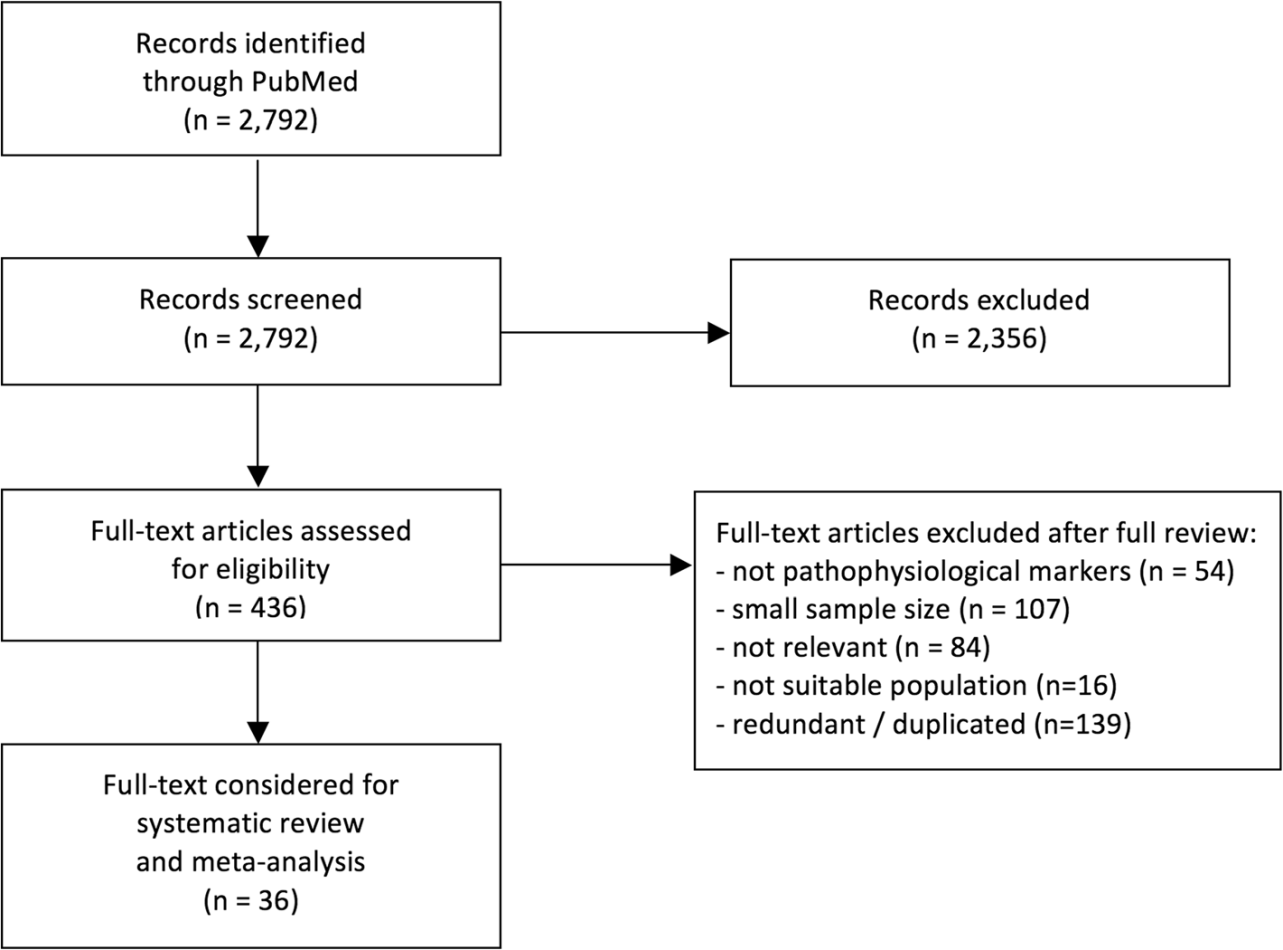
Flowchart for bibliographic search

We identified 2792 articles from the PubMed screen. Four-hundred and thirty-six full-text articles were assessed for eligibility, and 36 articles were included in the systematic review and meta-analyses (Fig. 1). These reports were representative of 6602 subjects with mean age ranging from 53 to 86 years. Thirty-three papers assessed preclinical AD in cognitively normal subjects, reporting data on 5537 subjects. Other papers assessed SCD (three studies, 280 subjects), SCI (one study, 60 subjects), and SMC (three studies, 725 subjects).

Amyloid-PET data were extracted from 20 studies. Of these, 14 studies used [^11^C]Pittsburgh compound-B (PiB) radiotracer, three used [^18^F]florbetapir, and three used [^18^F]florbetaben. CSF data were available in 15 studies. Of these, an AD-like profile (i.e., abnormal Aβ42, total tau, and p-tau) was reported in 14 studies and altered CSF Aβ42/t-Tau ratio in one study (see Additional file 2: Table S2).

Neuropsychological criteria for the definition of diagnostic groups were heterogeneous. Baseline characteristics according to cognitive criteria were CDR = 0 (12 studies, 3484 subjects), CDR = 0 plus Mini Mental State Examination (MMSE) score ≥ 27 (five studies, 1086 subjects), no neuropsychological deficits and not reaching MCI criteria (13 studies, 3297 subjects), score ≥ 23 on Montreal Cognitive Assessment (one study, 132 subjects), and cutoff value 1SD below the age-corrected normative mean in the neuropsychological battery (one study, 570 subjects).

Baseline criteria to define SCD were presence of self-report cognitive complaints (five studies, 765 subjects) and SCD-I criteria [39] (one study, 85 subjects). Only in one study [56] was SCD assessed by means of specific questionnaires (318 subjects). Baseline criteria to define subtle cognitive decline were applied only in four studies and were heterogeneous: > 1 SD below the age-corrected normative mean on two of six neuropsychological measures in different cognitive domains, Functional Assessment Questionnaire score ≥ 6 (one study, 50 subjects); − 1.25 SD at MMSE and one episodic memory composite score (one study, 13 subjects); lowest 10th percentile in a global cognitive summary score (one study, six subjects); and 1.5 SD less than mean score in one neuropsychological test obtained in normal controls (one study, six subjects).

Years of education (see Additional file 2: Table S3) and gender were included as covariates in meta-regression but they turned out to be not significantly associated with preclinical AD prevalence. Age was consistently associated with preclinical AD according to meta-regression results but this was not sufficient to explain heterogeneity. In fact, heterogeneity continued to remain substantial even when subgroup analyses were carried out according to the mean age of participants (see Additional file 3: Figure S1).

The characteristics of the studies included in the systematic review and meta-analysis are reported in Table 1. Since only two studies provided data on ethnicity, we have not included these data within the demographics. This, however, highlights the need for large studies paying attention to the inclusion of different ethnicities, in order to obtain results that may be generalized to the whole population.

**Table 1:** Characteristics of the cohorts examined

Reference	Cohort	Group	*N*	Gender (M/F)	Mean age (years)	Mean MMSE	Neuropsychological criteria	Biomarkers
Arenaza-Urquijo et al., 2017 [69]	IMAP+	CN	73	39/34	66.9	29.0	MMSE score ≥ 28	AV45-PET
Barthel et al., 2011 [77]	FBB phase 2 study	CN	68	30/38	68.2	NA	CDR = 0; MMSE score ≥ 28	FBB-PET
Besson et al., 2015 [78]	IMAP	CN	54	27/27	65.8	29.0	Cognitive performance > 5th percentile	AV45-PET
Brier et al., 2016 [79]	Washington University ACS-KADRC	CN	157	50/107	53.1	29.2	CDR = 0	PiB-PET
Byun et al., 2017 [80]	KBASE	CN	205	98/107	68.5	NA	CDR = 0	PiB-PET
Cho et al., 2016 [81]	Memory Clinic Gangnam Hospital	CN	67	25/42	66.1	28.1	No neuropsychological deficits	FBB-PET
Clark et al., 2018 [82]	WRAP	CN	314	96/218	61.5	NA	No neuropsychological deficits	CSF—NIA-AA criteria
Dubois et al., 2018 [56]	INSIGHT_preAD	CN	318	117/201	76.0	28.7	Cognitive complaints; MMSE score ≥ 27, CDR = 0, FCSRT total recall score ≥ 41	AV45-PET
Eckerström et al., 2017 [72]	Gothenburg MCI Study	SCD	113	37/76	62.0	28.0	Cognitive complaints (> 6 months)	CSF—NIA-AA criteria
Edmonds et al., 2015 [11]	ADNI	CN	570	308/262	73.0	NA	No neuropsychological deficits	CSF—NIA-AA criteria
Gordon et al., 2015 [68]	WU-KADRC	CN	397	141/257	67.1	29.2	CDR = 0	PiB-PET
Harrington et al., 2013 [83]	Huntington Hospital—Pasadena	CN	70	27/43	77.2	NA	CDR = 0; FAQ = 0; no neuropsychological deficits	CSF Aβ42/t-tau ratio
Hatashita and Yamasaki, 2010 [84]	Shonan Atsugi Hospital—Japan	CN	91	45/46	65.1	29.3	CDR = 0; MMSE score ≥ 28	PiB-PET
Johnson et al., 2013 [85]	AV45-A11 study	CN	78	34/44	69.4	29.6	MMSE score ≥ 29; no neuropsychological deficits	PiB-PET
Kern et al., 2018 [15]	H70 Gothenburg Birth Cohort Studies	CN	259	130/129	70.6	29.3	CDR = 0	CSF—NIA-AA criteria
Knopman et al., 2012 [73]	MCSA	CN	529	289/240	78.3	28	No neuropsychological deficits	PiB-PET
Lilamand et al., 2016 [86]	MAPT	CN	271	108/163	76.0	28.2	CDR = 0	PiB-PET
Lim et al., 2014 [87]	University of Pittsburgh ADRC and Pepper Registry	CN	56	19/37	75.8	28.5	CDR = 0; MMSE score > 27	PiB-PET
Lim et al., 2016 [31]	AIBL	CN	423	192/231	69.4	28.8	No neuropsychological deficits	PiB-PET
Mandecka et al., 2016 [58]	Cracow Hospital—Memory Clinic	SCD	85	28/57	61.3	NA	Cognitive complaints	CSF Aβ42 and t-tau
Meyer et al., 2018 [88]	PreventAD	CN	101	31/70	62.9	NA	MoCA ≥ 23	CSF—NIA-AA criteria
Montal et al., 2018 [89]	Spain cohorts	CN	254	141/113	58.6	28.9	No cognitive complaints; CDR = 0; no neuropsychological deficits	CSF—NIA-AA criteria
Ossenkoppele et al., 2014 [90]	BACS	CN	81	29/52	75.0	29.0	No cognitive complaints; no neuropsychological deficits	PiB-PET
Papp et al., 2017 [28]	HABS	CN	279	114/165	73.4	29.0	CDR = 0; no deficits on Logical Memory Story A, Delayed Recall, and MMSE	PiB-PET
Rodrigue et al., 2012 [91]	DLBS	CN	137	NA/NA	64.0	29.3	No neuropsychological deficits	AV45-PET
Schoonenboom et al., 2012 [92]	VU Medical Center, Alzheimer Center, Amsterdam	SMC	275	151/124	59.0	29.0	No neuropsychological deficits	CSF Aβ42,t-tau, p-tau
Snyder et al., 2016 [70]	Rhode Island and Alzheimer Assessment Trial Match	CN	63	24/39	62.8	29.1	MMSE score ≥ 27; no neuropsychological deficits	AV45-PET
Soldan et al., 2016 [26]	BIOCARD	CN	222	89/133	56.9	29.5	No neuropsychological deficits	CSF Aβ_42_, t-tau, p-tau
Taylor et al., 2017 [93]	APEX	CN	128	34/94	71.3	29.0	CDR = 0; no neuropsychological deficits	PiB-PET
Um et al., 2017 [94]	Catholic Geriatric Neuroimaging Database	CN	50	18/32	68.0	28.5	CDR = 0; MMSE score > 27	FBB-PET
Van Harten et al., 2013 [95]	Amsterdam Dementia Cohort	SMC	132	76/56	61.4	28.3	Cognitive complaints; no neuropsychological deficits	CSF—NIA-AA criteria
Visser et al., 2009 [59]	DESCRIPA	CN	89	41/48	67.1	29.3	No neuropsychological deficits	CSF Aβ42/tau
		SCI	60	31/29	66.0	28.8	Cognitive complaints; no neuropsychological deficits	CSF Aβ42/tau
Wolfsgruber et al., 2015 [96]	DCN	SCD	82	58/24	66.7	27.7	No neuropsychological deficits	CSF Aβ42/t-tau
Zhao et al., 2018 [57]	GEM	CN	175	104/71	86.0	NA	No neuropsychological deficits	PiB-PET

*Aβ* amyloid beta, *ACS-KADRC* Adult Children Study Knight Alzheimer’s Disease Research Center, *AD* Alzheimer’s disease, *ADNI* Alzheimer’s Disease Neuroimaging Initiative, *ADRC* Knight Alzheimer’s Disease Research Center; *AIBL* Australian Imaging Biomarkers & Lifestyle study, *APEX* University of Kansas’s Alzheimer’s Prevention through Exercise, *AV45* florbetapir, *BACS* Berkeley Aging Cohort Study, *BIOCARD* Biomarkers of Cognitive Decline Among Normal Individuals, *CDR* Clinical Dementia Rating Scale, *CN* cognitively normal, *CSF* cerebrospinal fluid, *DESCRIPA* Development of screening guidelines and criteria for pre-dementia Alzheimer’s disease, *DCN* German Dementia Competence Network, *DLBS* Dallas Lifespan Brain Study, *F* female, *FAQ* Functional Assessment Questionnaire, *FBB* florbetaben, *FCSRT* Free And Cued Selective Reminding Test, *GEM* Ginkgo Evaluation of Memory, *HABS* Harvard Aging Brain Study, *IMAP* Imagerie Multimodale de la Maladie d’Alzheimer à un stade Precoce, *INSIGHT_preAD* Investigation of Alzheimer’s Predictors in Subjective Memory Complainers, *KBASE* Korean Brain Aging Study for Early Diagnosis and Prediction of Alzheimer’s Disease, *M* male, *MAPT* Multidomain Alzheimer Preventive Trial, *MCI* mild cognitive impairment, *MCSA* Mayo Clinic Study of Aging, *MMSE* Mini Mental State Examination, *MoCA* Montreal Cognitive Assessment, *NIA-AA* National Institute on Aging and Alzheimer’s Association, *NA* not assessed, *PET* positron emission tomography, *PiB* Pittsburgh compound, *p-tau* phosphorylated tau, *t-tau* total tau, *SCD* subjective cognitive decline, *SCI* subjective cognitive impairment, *SMC* subjective memory complaints, *WRAP* Wisconsin Registry for Alzheimer’s Prevention, *WU-KADRC* Knight Alzheimer’s Disease Research Center at Washington University

After evaluating the risk of bias, we judged that 14 studies were at low risk of bias, 21 at moderate risk, and none at high risk. According to the risk of bias assessment, we found that in most of the included studies this was based on enrollment procedures that do not ensure a true or close representation of the target population (*n* = 26). In many cases, the study’s population was not representative of the national population (*n* = 25) and almost all of the studies were conducted without random sampling or census. Detailed results are reported in Additional file 4: Table S4).

**Fig. 2:**
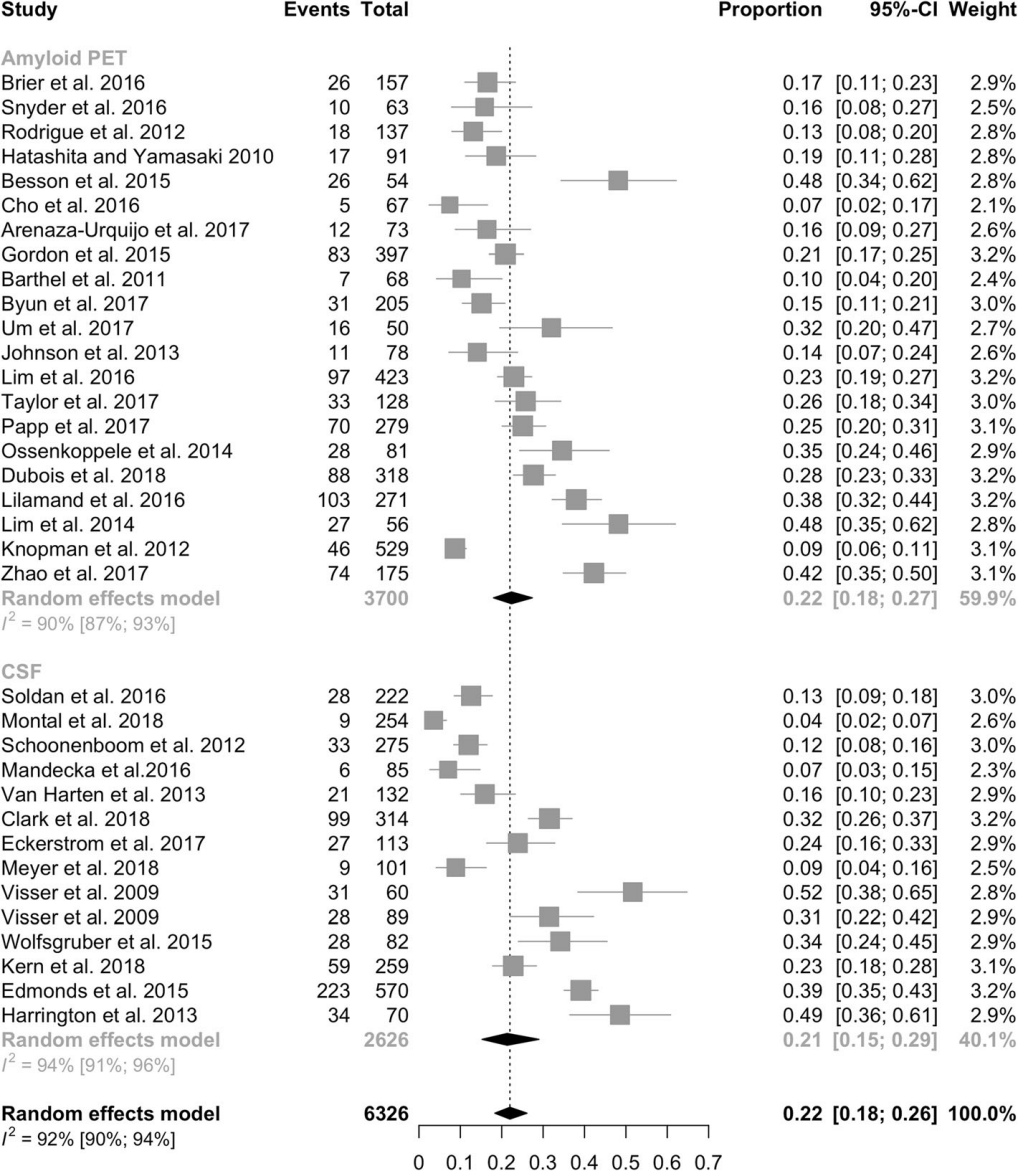
Prevalence of pathophysiological biomarker positivity across the 2011 NIA-AA preclinical AD stages. CI confidence interval, CSF cerebrospinal fluid, PET positron emission tomography

We first considered evidence of abnormalities in the AD diagnostic markers (CSF AD profile, either with the three biomarkers altered [amyloid-β1–42 plus t-tau and p-tau] or Aβ42/tau ratio, or amyloid PET positivity) in cognitively healthy subjects, so defined as preclinical AD (Fig. 2). Preclinical AD was documented in 22% of cognitively normal individuals (95% CI = 18–26%, *I*^2^ = 92%). Prevalence was dependent on age, as tested with meta-regression, ranging from 16.5% at 53 years to 53% at 86 years [57], with an increase in prevalence of 1.0% per year (95% CI = 0.5–5%). According to CSF analysis, preclinical AD was found in 21% of individuals (95% CI = 15–29%, *I*^2^ = 94%), as compared to 22% (95% CI = 18–27%, *I*^2^ = 90%) when considering amyloid-PET positivity.

Prevalence of preclinical AD in individuals with subjective cognitive decline was analogous to that documented in normal subjects (23%, 95% CI = 14–33%, *I*^2^ = 92%), ranging from 7% [58] to 52% [59].

### Prevalence of preclinical AD stages according to 2011 NIA-AA criteria

**Fig. 3:**
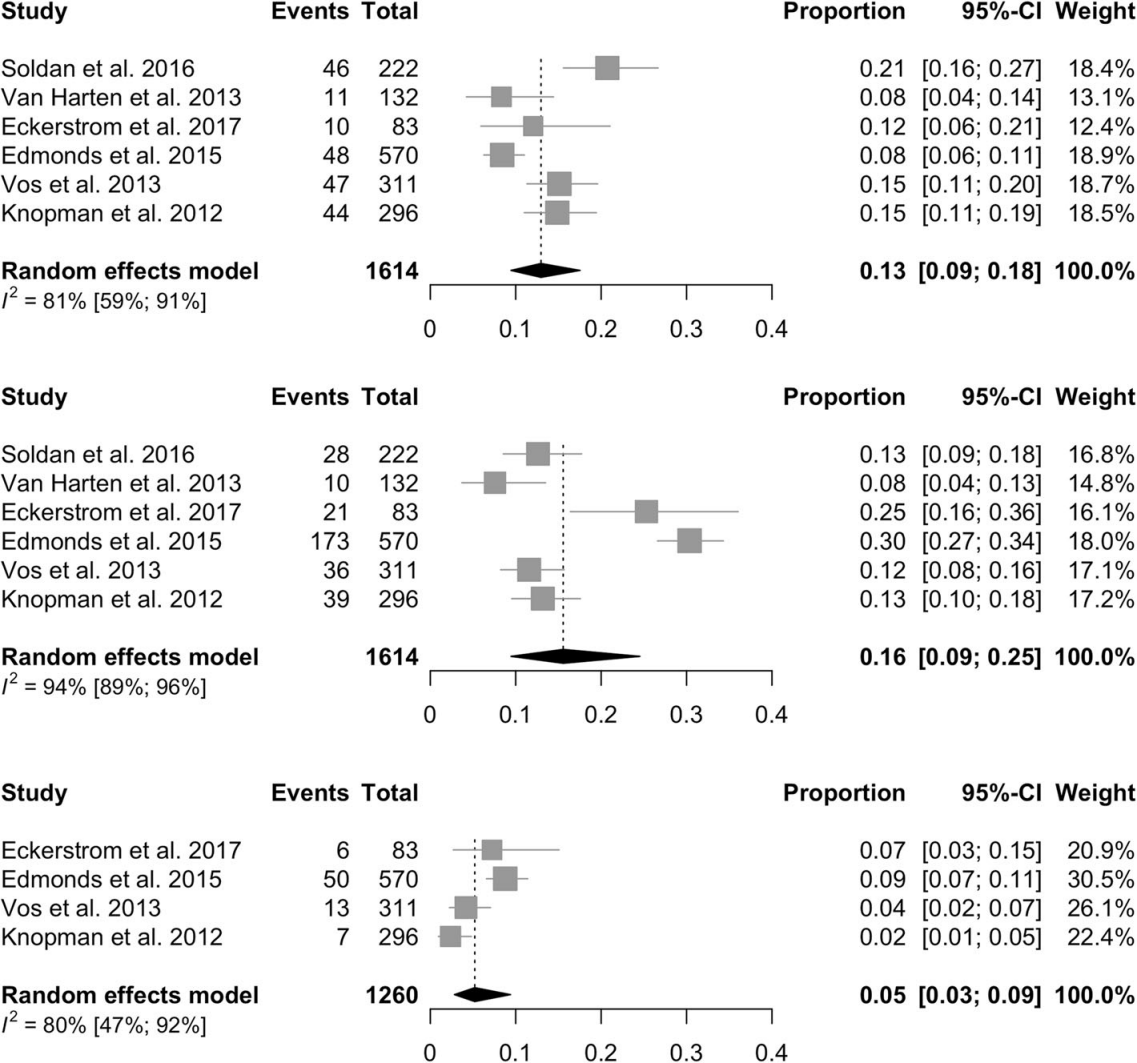
Prevalence of preclinical AD stages according to 2011 NIA-AA criteria. CI confidence interval

We calculated the prevalence of preclinical AD stages, defined according to 2011 NIA-AA criteria, in six studies published after 2011, between 2011 and 2018 (Fig. 3). Prevalence of Stage 1 was 13% (95% CI = 9–18%, *I*^2^ = 81%), of Stage 2 was 16% (95% CI = 9–25%, *I*^2^ = 94%), and of Stage 3 was 5% (95% CI = 3–9%, *I*^2^ = 80%).

### Clinical progression

**Fig. 4:**
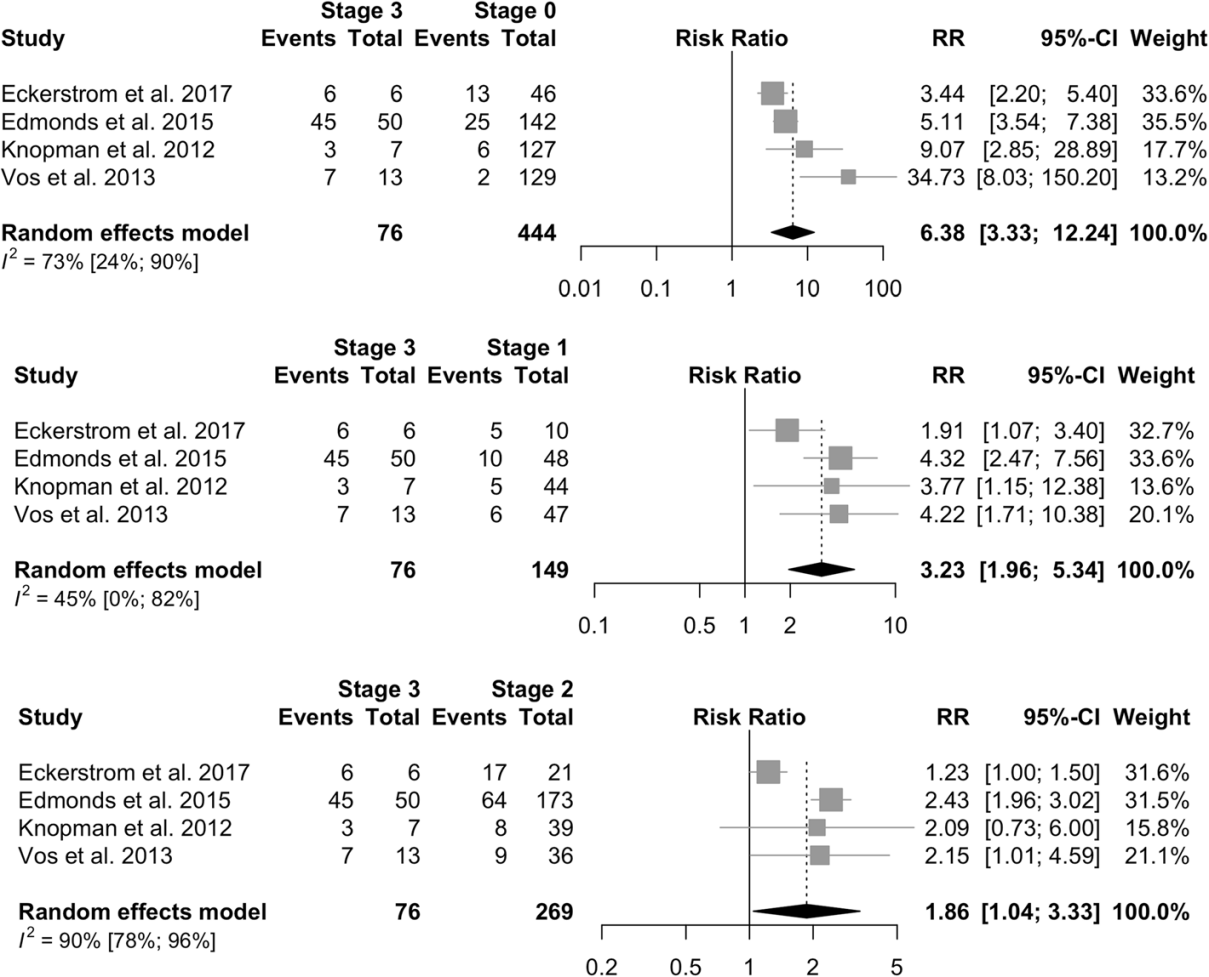
Relative risk of progression among 2011 NIA-AA preclinical AD stages. CI confidence interval, RR relative risk

Positivity of biomarkers confers a higher relative risk of clinical progression as measured by conversion to MCI or AD (RR = 2.75, 95% CI = 2.32–3.27, *I*^2^ = 67%). The risk of clinical progression is analogous in cognitively normal subjects (RR = 2.65, 95% CI = 2.18–3.23, *I*^2^ = 53%) and in subjects with SCD, SCI, or SMC (RR = 3.30, 95% CI = 2.41–4.53, *I*^2^ = 87%). Rates of clinical progression across the 2011 NIA-AA preclinical AD stages are presented in Table 2. The risk of progression increases across preclinical AD stages, with individuals classified as NIA-AA Stage 3 showing the highest risk (73%, 95% CI = 40–92%) compared to those in Stage 2 (38%, 95% CI = 21–59%) and Stage 1 (20%, 95% CI = 10–34%). The clinical progression rate in Stage 3 was significantly higher as compared to subjects classified as having normal biomarkers (RR = 6.38, 95% CI = 3.33–12.24, *I*^2^ = 73%; Fig. 4), in Stage 1 (RR = 3.23, 95% CI = 1.96–5.34, *I*^2^ = 45%), and in Stage 2 (RR = 1.86, 95% CI = 1.04–3.33, *I*^2^ = 90%).

**Table 2:** Rates of clinical progression across NIA-AA preclinical AD stages

Reference	Cohort	Group	*N*	Clinical progression	Mean follow-up (years)	Stage 0, % (*n* progr/*n* tot)	Stage 1, % (*n* progr/*n* tot)	Stage 2, % (*n* progr/*n* tot)	Stage 3, % (*n* progr/*n* tot)
Eckerström et al., 2017 [72]	Gothenburg MCI Study	CN	113	Cognitive decline–dementia^a^	4	28% (13/46)	50% (5/10)	81% (17/21)	100% (6/6)
Edmonds et al., 2015 [11]	ADNI	CN	570	MCI–dementia^b^	2.7	18% (25/142)	21% (10/48)	37% (64/173)	90% (45/50)
Knopman et al., 2012 [73]	MCSA	CN	296	MCI–dementia^b^	1.3	5% (6/127)	11% (5/44)	21% (8/39)	43% (3/7)
Soldan et al., 2016 [26]	BIOCARD	CN	222	MCI–dementia^b^	10.4	18% (18/102)	^19.5^20% (9/46)	54% (15/28)	Not specifically addressed
Van Harten et al., 2013 [95]	Amsterdam Dementia Cohort	SMC	132	MCI–dementia^b^	1.5	3% (2/80)	18% (2/11)	60% (6/10)	Not specifically addressed
Vos et al., 2013 [62]	WU-ADRC	CN	311	MCI^c^	3.4	2% (2/129)	13% (6/47)	25% (9/36)	54% (7/13)

*AD* Alzheimer’s disease, *ADNI* Alzheimer’s Disease Neuroimaging Initiative, *BIOCARD* Biomarkers of Cognitive Decline Among Normal Individuals, *CN* cognitively normal, *MCI* mild cognitive impairment, *MCSA* Mayo Clinic Study of Aging, *NIA-AA* National Institute on Aging and Alzheimer’s Association, *SMC* subjective memory complaints, n *progr*/n *tot* number progressed/total number, *WU-ADRC* Washington University Alzheimer’s Disease Research Center

^a^Cognitive decline outcome defined as decline in neuropsychological test results or to clinical dementia (using Global Deterioration Scale and criteria for dementia), at follow-up

^b^Progression to diagnosis of MCI and dementia due to AD (NIA-AA criteria)

^c^Progression to Clinical Dementia Rating Scale of at least 0.5 (MCI), symptomatic AD (score of at least 0.5 for memory and at least one other domain and cognitive impairments deemed to be due to AD)

## Discussion

The primary aim of this systematic review was to evaluate whether preclinical AD Stage 3 might rather be considered the clinical debut of AD. This conceptual change would imply that in clinical practice we should actively look for it as a specific clinical entity. To do this, all of the existing data from studies assessing the prevalence of AD pathophysiological biomarker positivity (CSF, amyloid PET) in the whole spectrum preceding MCI (cognitively normal subjects, subjective cognitive decline, subtle impairment not fulfilling MCI criteria, *n* = 6602), as well as their evolution toward cognitive worsening/dementia, were analyzed. In the cohorts examined, along the preclinical AD phases, mean prevalence of Stage 3 is 5%, similar to what was found in the ADNI cohort, in which the prevalence of Stage 3 ranges from 2% up to 9% [11]. In this stage, according to available data (different cohorts followed up for 1–4 years), the pooled risk of progression was 73% (95% CI = 40–92%); that is, individuals show, on average, a 6-fold increased risk of progression (relative risk ranging from 3.4 to 34.7; Fig. 4) compared to those with normal biomarkers (Stage 0). These figures are quite similar to those observed for MCI due to AD, which shows a 3-year progression rate to dementia of around 60%, with a hazard ratio of 14 (95% CI = 5.9–35.2) [60]. Overall positivity of pathophysiological biomarkers was similar between amyloid PET (22%) and CSF (25%). Differences in rates of positivity in CSF vs amyloid PET in CN range from 8 to 21% [61–64]. CN and SCD groups do not differ in rates of biomarker positivity, possibly due to the unclear differentiation between these categories.

An AD-like CSF profile, as well as a positive amyloid PET, is not rarely observed in cognitively normal individuals [65], and such positivity increases with age [65–67]. No longitudinal data assessing these subjects for an extended time window (> 10 years) are available, therefore we cannot take into account the actual value of these data as a matter of preclinical AD.

An important issue in our systematic review concerns the different methods applied for cognitive characterization of CN individuals, and this could explain the heterogeneity in prevalence of pathophysiological biomarker positivity and risk of progression. Some studies used a CDR score of 0 [12, 68], other studies report the MMSE scores [69, 70], and other investigations refer to a condition not fulfilling MCI criteria, without any specific definition [71]. In the clinical setting, subtle, although detectable, cognitive decline in a subject who expresses the will to learn about the cause of her/his impairment may justify biomarker assessment. At present, neuropsychological criteria defining “subtle” cognitive decline are not yet available [11, 63]. According to Epelbaum et al. [24], different cutoff scores might be used, namely a performance below 1.5 SD in two out of six cognitive measures [72], or below 1 SD in one neuropsychological test [11, 24], or performances falling below the 10th percentile [8, 73]. The MMSE is not appropriate for detecting subtle symptoms. Therefore, more sensitive cognitive measures should be used, namely composite scores, which are also able to track subtle decline over time. Since subtle cognitive decline has been considered either as cognitive changes over time, in longitudinal cohorts, or poorer scores at baseline, in cross-sectional studies [12], it is necessary to further address this issue in ad-hoc studies. These data confirm how difficult it is to define the boundary between normal cognition and subtle cognitive deficits, and measurement of the change with time might be the right option, in terms of prevention/early diagnosis programs.

The lack of consensus about definition of CN individuals may lead to different preclinical AD rates. Accordingly, cognitively normal individuals in the cohorts we examined could have been included either as volunteers—classified as healthy controls in research studies—or considered as a control group in post-hoc analysis, when cross-sectional studies were included (i.e., ADNI cohorts). These two different criteria for subject selection may actually reflect two distinct populations. Such a difference can lead to noncomparable findings.

Importantly, the analyses indicated a great heterogeneity in SCD definition. Up to now, although an attempt to implement SCD research criteria toward a harmonization of SCD measures has been recently carried out [46], no common criteria are available [33, 43, 56, 74]. Only one study followed SCD-I [39, 58], another investigation considered the presence of complaints about memory [75], and only one investigation used a specific questionnaire to objectify the complaints [56]. A possible explanation could be the wide time window (2008–2018) we considered for paper selection, reflecting the lexical evolution of SCD as an entity, according to SCD-I criteria [39]. Recent work by Opdebeeck et al. [76] found that different methods to assess SCD may lead to different conclusions about the rates of risk progression explaining discrepancies in the predictive value of SCD among studies. Therefore, longitudinal studies about SCD are needed to understand the role and predictive value of this entity. According to Sperling et al. [2], SCD is not a mandatory step along the preclinical AD stages [72].

With respect to the diagnostic equivalence of pathophysiological biomarkers, consistent evidence shows that CSF biomarkers and amyloid PET are comparable in detecting the AD signature [61]. CSF biomarkers have the advantage to give simultaneous information about the presence of amyloidosis (Aβ42, Aβ42/40), tauopathy (p-tau), and neurodegeneration (total tau). According to the recent NIA-AA Research Framework [14], in order to diagnose AD, the positivity of both markers of brain amyloidosis (A^+^) and markers of tauopathy (T^+^) is needed, regardless of the clinical stage.

## Conclusions

According to the available data in terms of prevalence and risk of progression of 2011 NIA-AA preclinical AD Stage 3, subtle cognitive decline associated with pathophysiological AD biomarker positivity likely represents the earliest symptomatic phase of AD, thus belonging to clinical, rather than preclinical, AD ("pre-MCI due to AD"). Accordingly, individuals belonging to 2011 NIA-AA Stage 3 (i.e., showing subtle cognitive decline and positivity to pathophysiological markers of AD) should be considered in the same way as the category MCI due to AD. This allocation to the clinical phase of AD would allow the clinician to timely include the patient in secondary/tertiary prevention treatment. Before adoption of "pre-MCI due to AD" in routine clinical use, this diagnostic entity deserves to be fully validated with pathological studies as well as with ad-hoc prospective longitudinal observations.

## Additional files

Additional file 1: **Table S1.** PRISMA Checklist. (DOC 63 kb)

Additional file 2: **Table S2.** Cut-offs used for defining pre-clinical Alzheimer’s disease according biomarkers positivity. **Table S3.** Education years. (DOCX 53 kb)

Additional file 3: **Figure S1.** Subgroup analysis according to mean age of participants. (PNG 1040 kb)

Additional file 4: **Table S4.** Risk of Bias Assessment. (DOCX 45 kb)
