# Supplementary material for: Prevalence and risk of progression of preclinical Alzheimer’s disease stages: a systematic review and meta-analysis
Source: Alzheimers Res Ther. 2019 Jan 15;11:7. doi: 10.1186/s13195-018-0459-7 (PMC6334406; doi:10.1186/s13195-018-0459-7)
Supplement: Supplementary file 2 — Table S2. Cut-offs used for defining pre-clinical Alzheimer’s disease according biomarkers positivity. Table S3. Education years. (DOCX 53 kb) [file 13195_2018_459_MOESM2_ESM.docx]

**Table S2 –** Cut-offs used for defining pre-clinical Alzheimer’s disease according biomarkers positivity

| **Reference** | **Cohort** | **Group** | **Biomarkers** | **Biomarkers cut off** |
| --- | --- | --- | --- | --- |
| Arenaza-Urquijo et al. 2017 | IMAP + | CN | AV45-PET | NA |
| Barthel et al. 2011 | FBB phase 2 study | CN | FBB-PET | Global / regional BAPL 1-3 |
| Besson et al. 2015 | IMAP | CN | AV45-PET | SUVR ≥ 1.005 |
| Brier et al. 2016 | Washington University ACS-KADRC | CN | PiB-PET | SUVR ≥ 1.42 |
| Byun et al. 2017 | KBASE | CN | PiB-PET | SUVR ≥ 1.40 |
| Cho et al. 2016 | Memory Clinic Gangnam Hospital | CN | FBB-PET | SUVR ≥ 1.40 |
| Clark et al. 2018 | WRAP | CN | CSF- NIA-AA criteria | Aβ42/Aβ40 ≤ 0.009 pg/mL  t -Tau ≥ 461,26 pg/mL  p-Tau≥ 59,5 pg/mL |
| Dubois et al. 2018 | INSIGHT_preAD | CN | AV45-PET | SUVR ≥ 0.79 |
| Eckerstrom et al. 2017 | Gothenburg MCI study | SCD | CSF- NIA-AA criteria | Aβ42 ≤ 482 pg/mL  t -Tau ≥ 320 pg/mL  p-Tau≥ 52 pg/mL |
| Edmonds et al. 2015 | ADNI | CN | CSF- NIA-AA criteria | Aβ42 ≤ 192 pg/mL  t -Tau ≥ 93 pg/mL  p-Tau≥ 23 pg/mL |
| Gordon et al. 2015 | WU-KADRC | CN | PiB-PET | SUVR ≥ 0.18 |
| Harrington et al. 2013 | Huntington Hospital-Pasadena | CN | CSF Aβ₄₂/t-tau ratio | Aβ42 ≤ 550 pg/mL  t -Tau ≥ 500 pg/mL  p-Tau≥ 80 pg/mL |
| Hatashita and Yamasaki 2010 | Shonan Atsugi Hospital-Japan | CN | PiB-PET | SUVR ≥ 1.49 |
| Johnson et al. 2013 | AV45-A11 study | CN | PiB-PET | SUVR ≥ 1.10 |
| Kern et al. 2018 | H70 Gothenburg Birth Cohort Studies | CN | CSF- NIA-AA criteria | Aβ42 ≤ 530 pg/mL  t -Tau ≥ 350 pg/mL  p-Tau≥ 80 pg/mL |
| Knopman et al. 2012 | MCSA | CN | PiB-PET | SUVR ≥ 1.5 |
| Lilamand et al. 2016 | MAPT | CN | PiB-PET | SUVR ≥ 1.17 |
| Lim et al. 2014 | University of Pittsburgh ADRC and Pepper Registry | CN | PiB-PET | ACC_SUVR ≥ 1,78  FC_SUVR ≥ 1,71  LTC_SUVR ≥ 1,50  MTC_SUVR ≥ 1,42  PC_SUVR ≥ 1,63  PC_SUVR ≥ 1,73 |
| Lim et al. 2016 | AIBL | CN | PiB-PET | SUVR ≥ 1.5 |
| Mandecka et al. 2016 | Cracow Hospital-Memory Clinic | SCD | CSF Aβ₄₂ and t-tau | Aβ42 ≤ 609.54 pg/mL  t -Tau ≥ 277.02 pg/mL  p-Tau≥ 55.08 pg/mL |
| Meyer et al. 2018 | PreventAD | CN | CSF- NIA-AA criteria | Aβ42 ≤ 870 pg/mL  t -Tau ≥ 355 pg/mL |
| Montal et al. 2018 | Spain cohorts | CN | CSF- NIA-AA criteria | Aβ42 ≤ 550 pg/mL  p -Tau ≥ 61 pg/mL |
| Ossenkoppele et al. 2014 | BACS | CN | PiB-PET | NA |
| Papp et al. 2017 | HABS | CN | PiB-PET | SUVR≥1.20 |
| Rodrigue et al. 2012 | DLBS | CN | AV45-PET | SUVR ≥ 1.22 |
| Schoonenboom et al. 2012 | VU Medical Center, Alz Center, Amsterdam | SMC | CSF Aβ₄₂,t-tau, p-tau | score below 1 using the formula:  CSFAβ42/152+8.25 * p-tau |
| Snyder et al. 2016 | Rhode Island and Alz Ass Trial Match | CN | AV45-PET | SUVR ≥ 1.1 |
| Soldan et al. 2016 | BIOCARD | CN | CSF Aβ₄₂, t-tau, p-tau | Aβ42 ≤ 374.5 pg/mL  t -Tau ≥ 74.9 pg/mL  p-Tau≥39.4 pg/mL |
| Taylor et al. 2017 | APEX | CN | PiB-PET | SUVR≥1.18 |
| Um et al. 2017 | Cath Ger Neuroimaging Database | CN | FBB-PET | NA |
| Van Harten et al. 2013 | Amsterdam Dementia Cohort | SMC | CSF - NIA-AA criteria | Aβ42 ≤ 550 pg/mL  t -Tau ≥ 375 pg/mL  p-Tau≥ 52 pg/mL |
| Visser et al. 2009 | DESCRIPA | CN | CSF Aβ₄₂/tau | score below 1 using the formula:  CSFAβ42/(240+[1.18 * T-tau]) |
|  |  | SCI | CSF Aβ₄₂/tau | score below 1 using the formula:  CSFAβ42/(240+[1.18 * T-tau]) |
| Wolfsgruber et al. 2015 | DCN | SCD | CSF Aβ₄₂/t-tau | Aβ42 ≤ 600 pg/mL  t -Tau ≥ 300 pg/mL  p-Tau≥ 60 pg/mL |
| Zhao et al. 2017 | GEM | CN | PiB-PET | SUVR≥1.57 |

ACS-KADRC: Adult Children Study Knight Alzheimer’s Disease Research Center; AD: Alzheimer’s Disease; ADNI: Alzheimer’s Disease Neuroimaging Initiative; ADRC: Knight Alzheimer’s Disease Research Center; AIBL: Australian Imaging Biomarkers & Lifestyle study; APEX University of Kansas's Alzheimer's Prevention through Exercise: AV45:Florbetapir; BACS: Berkeley Aging Cohort Study; BIOCARD: Biomarkers of Cognitive Decline Among Normal Individuals; CN: Cognitively Normal; CSF: cerebrospinal fluid; DESCRIPA: Development of screening guidelines and criteria for pre-dementia Alzheimer’s disease; DCN: German Dementia Competence Network; DLBS: Dallas Lifespan Brain Study; FBB: Florbetaben; GEM: Ginkgo Evaluation of Memory; HABS: Harvard Aging Brain Study IMAP: Imagerie Multimodale de la Maladie d’Alzheimer à un stade Precoce; INSIGHT_preAD: Investigation of Alzheimer’s Predictors in Subjective Memory Complainers; KBASE: Korean Brain Aging Study for Early Diagnosis and Prediction of Alzheimer’s Disease; MAPT: Multidomain Alzheimer Preventive Trial; MCSA: Mayo Clinic Study of Aging; NIA-AA: National Institute on Aging and Alzheimer's Association; NA: not assessed; PET: Positron Emission Tomography; PiB: Pittsburgh compound; p-tau: Phosphorylated Tau; t-tau: Total Tau; SCD: Subjective Cognitive Decline; SMC: Subjective Memory Complaints; WRAP: Wisconsin Registry for Alzheimer's Prevention; WU-ADRC: Washington University Alzheimer's Disease Research Center; WU-KADRC: Knight Alzheimer’s Disease Research Center at Washington University

**Table S3 –** Education years

| **Reference** | **Cohort** | **Group** | **Education years** |
| --- | --- | --- | --- |
| Arenaza-Urquijo et al. 2017 | IMAP + | CN | 12.2±3.8 |
| Barthel et al. 2011 | FBB phase 2 study | CN | 14.7±3.9 |
| Besson et al. 2015 | IMAP | CN | 11.7±3.7 |
| Brier et al. 2016 | Washington University ACS-KADRC | CN | 15.6±2.4 |
| Byun et al. 2017 | KBASE | CN | 12±4.8 |
| Cho et al. 2016 | Memory Clinic Gangnam Hospital | CN | 11.8±4.4 |
| Clark et al. 2018 | WRAP | CN | 16.3 |
| Dubois et al. 2018 | INSIGHT_preAD | CN | N.A. |
| Eckerstrom et al. 2017 | Gothenburg MCI study | SCD | 11±3 |
| Edmonds et al. 2015 | ADNI | CN | 16.4±2.74 |
| Gordon et al. 2015 | WU-KADRC | CN | N.A. |
| Harrington et al. 2013 | Huntington Hospital-Pasadena | CN | 6.25±1.9 |
| Hatashita and Yamasaki 2010 | Shonan Atsugi Hospital-Japan | CN | N.A. |
| Johnson et al. 2013 | AV45-A11 study | CN | 15.23±2.32 |
| Kern et al. 2018 | H70 Gothenburg Birth Cohort Studies | CN | 12.4±3.5 |
| Knopman et al. 2012 | MCSA | CN | 14 |
| Lilamand et al. 2016 | MAPT | CN | N.A. |
| Lim et al. 2014 | University of Pittsburgh ADRC and Pepper Registry | CN | 14.5±2.4 |
| Lim et al. 2016 | AIBL | CN | N.A. |
| Mandecka et al. 2016 | Cracow Hospital-Memory Clinic | SCD | 15.13±3.13 |
| Meyer et al. 2018 | PreventAD | CN | 14.88±2.93 |
| Montal et al. 2018 | Spain cohorts | CN | NA |
| Ossenkoppele et al. 2014 | BACS | CN | 17±1.8 |
| Papp et al. 2017 | HABS | CN | 15.85±3.04 |
| Rodrigue et al. 2012 | DLBS | CN | N.A. |
| Schoonenboom et al. 2012 | VU Medical Center. Alz Center, Amsterdam | SMC | N.A. |
| Snyder et al. 2016 | Rhode Island and Alz Ass Trial Match | CN | 17.21±2.77 |
| Soldan et al. 2016 | BIOCARD | CN | 17.2±2.3 |
| Taylor et al. 2017 | APEX | CN | 16.5±2.6 |
| Um et al. 2017 | Cath Ger Neuroimaging Database | CN | 9.55±2.25 |
| Van Harten et al. 2013 | Amsterdam Dementia Cohort | SMC | 6 |
| Visser et al. 2009 | DESCRIPA | CN | NA |
|  |  | SCI | 11.8±4.1 |
| Wolfsgruber et al. 2015 | DCN | SCD | 12.3±2.8 |
| Zhao et al. 2017 | GEM | CN | 14.7±2.6 |

ACS-KADRC: Adult Children Study Knight Alzheimer’s Disease Research Center; AD: Alzheimer’s Disease; ADNI: Alzheimer’s Disease Neuroimaging Initiative; ADRC: Knight Alzheimer’s Disease Research Center; AIBL: Australian Imaging Biomarkers & Lifestyle study; APEX University of Kansas's Alzheimer's Prevention through Exercise: AV45:Florbetapir; BACS: Berkeley Aging Cohort Study; BIOCARD: Biomarkers of Cognitive Decline Among Normal Individuals; CN: Cognitively Normal; CSF: cerebrospinal fluid; DESCRIPA: Development of screening guidelines and criteria for pre-dementia Alzheimer’s disease; DCN: German Dementia Competence Network; DLBS: Dallas Lifespan Brain Study; FBB: Florbetaben; GEM: Ginkgo Evaluation of Memory; HABS: Harvard Aging Brain Study IMAP: Imagerie Multimodale de la Maladie d’Alzheimer à un stade Precoce; INSIGHT_preAD: Investigation of Alzheimer’s Predictors in Subjective Memory Complainers; KBASE: Korean Brain Aging Study for Early Diagnosis and Prediction of Alzheimer’s Disease; MAPT: Multidomain Alzheimer Preventive Trial; MCSA: Mayo Clinic Study of Aging; SCD: Subjective Cognitive Decline; SMC: Subjective Memory Complaints; WRAP: Wisconsin Registry for Alzheimer's Prevention; WU-ADRC: Washington University Alzheimer's Disease Research Center; WU-KADRC: Knight Alzheimer’s Disease Research Center at Washington University
