# Supplementary figures and images for: Prevalence and risk of progression of preclinical Alzheimer’s disease stages: a systematic review and meta-analysis
Source: Alzheimers Res Ther. 2019 Jan 15;11:7. doi: 10.1186/s13195-018-0459-7 (PMC6334406; doi:10.1186/s13195-018-0459-7)

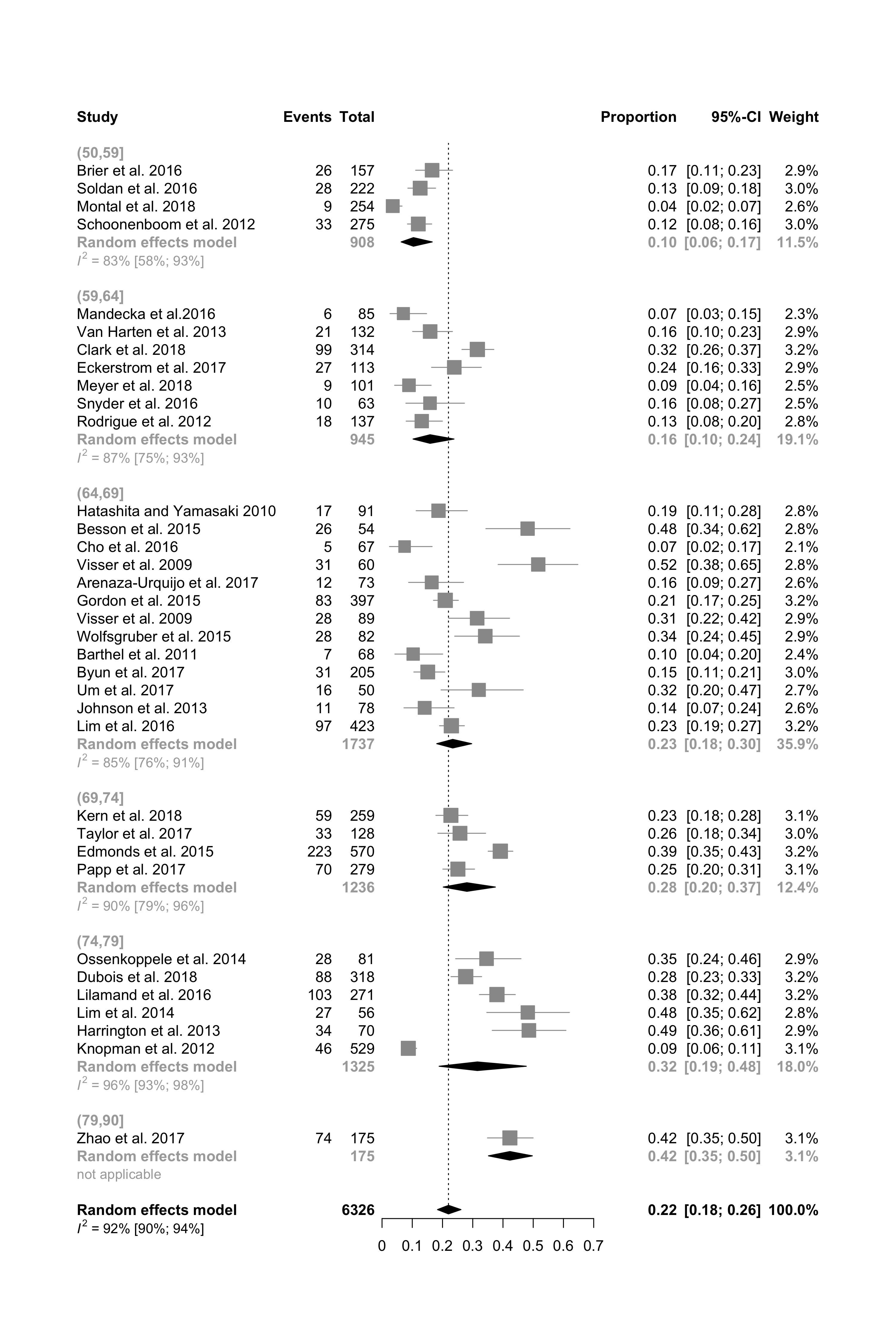

Supplement: Supplementary file 3 — Figure S1. Subgroup analysis according to mean age of participants. (PNG 1040 kb) [file 13195_2018_459_MOESM3_ESM.png]
