# Supplementary material for: Prevalence and risk of progression of preclinical Alzheimer’s disease stages: a systematic review and meta-analysis
Source: Alzheimers Res Ther. 2019 Jan 15;11:7. doi: 10.1186/s13195-018-0459-7 (PMC6334406; doi:10.1186/s13195-018-0459-7)
Supplement: Supplementary file 4 — Table S4. Risk of Bias Assessment. (DOCX 45 kb) [file 13195_2018_459_MOESM4_ESM.docx]

**Table S4 –** Risk of Bias Assessment

Hoy D, Brooks P, Woolf A, Blyth F, March L, Bain C, et al. Assessing risk of bias in prevalence studies: Modification of an existing tool and evidence of interrater agreement.

J Clin Epidemiol. Elsevier Inc; 2012;65:934–9

| **Reference** | **RoB 1** | **RoB 2** | **RoB 3** | **RoB 4** | **RoB 5** | **RoB 6** | **RoB 7** | **RoB 8** | **RoB 9** | **RoB score** | **RoB category** |
| --- | --- | --- | --- | --- | --- | --- | --- | --- | --- | --- | --- |
| Arenaza-Urquijo et al. 2017 | 1 | 1 | 1 | 1 | 0 | 0 | 0 | 0 | 0 | 4 | Moderate |
| Barthel et al. 2011 | 1 | 1 | 1 | 1 | 0 | 0 | 0 | 0 | 0 | 4 | Moderate |
| Besson et al. 2015 | 0 | 1 | 1 | 1 | 0 | 0 | 0 | 0 | 0 | 3 | Low |
| Brier et al. 2016 | 1 | 1 | 1 | 1 | 0 | 0 | 0 | 0 | 0 | 4 | Moderate |
| Byun et al. 2017 | 0 | 1 | 1 | 1 | 0 | 0 | 0 | 0 | 0 | 3 | Low |
| Cho et al. 2016 | 1 | 1 | 1 | 1 | 0 | 0 | 0 | 0 | 0 | 4 | Moderate |
| Clark et al. 2018 | 1 | 0 | 1 | 1 | 0 | 0 | 0 | 0 | 0 | 3 | Low |
| Dubois et al. 2018 | 0 | 0 | 1 | 1 | 0 | 0 | 0 | 0 | 0 | 2 | Low |
| Eckerstrom et al. 2017 | 0 | 1 | 1 | 1 | 0 | 0 | 0 | 0 | 0 | 3 | Low |
| Edmonds et al. 2015 | 1 | 1 | 1 | 1 | 0 | 0 | 0 | 0 | 0 | 4 | Moderate |
| Gordon et al. 2015 | 1 | 1 | 1 | 1 | 0 | 0 | 0 | 0 | 0 | 4 | Moderate |
| Harrington et al. 2013 | 0 | 1 | 1 | 1 | 0 | 0 | 0 | 0 | 0 | 3 | Low |
| Hatashita and Yamasaki 2010 | 1 | 1 | 1 | 1 | 0 | 0 | 0 | 0 | 0 | 4 | Moderate |
| Johnson et al. 2013 | 1 | 1 | 1 | 1 | 0 | 0 | 0 | 0 | 0 | 4 | Moderate |
| Kern et al. 2018 | 1 | 0 | 1 | 1 | 0 | 0 | 0 | 0 | 0 | 3 | Moderate |
| Knopman et al. 2012 | 0 | 0 | 0 | 1 | 0 | 0 | 0 | 0 | 0 | 1 | Low |
| Lilamand et al. 2016 | 0 | 1 | 1 | 1 | 0 | 0 | 0 | 0 | 0 | 3 | Low |
| Lim et al. 2014 | 1 | 1 | 1 | 1 | 0 | 0 | 0 | 0 | 0 | 4 | Moderate |
| Lim et al. 2016 | 1 | 1 | 1 | 1 | 0 | 0 | 0 | 0 | 0 | 4 | Moderate |
| Mandecka et al. 2016 | 1 | 1 | 1 | 1 | 0 | 0 | 0 | 0 | 0 | 4 | Moderate |
| Meyer et al. 2018 | 1 | 1 | 1 | 1 | 0 | 0 | 0 | 0 | 0 | 4 | Moderate |
| Montal et al. 2018 | 1 | 1 | 1 | 1 | 0 | 0 | 0 | 0 | 0 | 4 | Moderate |
| Ossenkoppele et al. 2014 | 1 | 1 | 1 | 1 | 0 | 0 | 0 | 0 | 0 | 4 | Moderate |
| Papp et al. 2017 | 1 | 1 | 1 | 1 | 0 | 0 | 0 | 0 | 0 | 4 | Moderate |
| Rodrigue et al. 2012 | 1 | 1 | 1 | 1 | 0 | 0 | 0 | 0 | 0 | 4 | Moderate |
| Schoonenboom et al. 2012 | 1 | 0 | 1 | 1 | 0 | 0 | 0 | 0 | 0 | 3 | Low |
| Snyder et al. 2016 | 1 | 1 | 1 | 1 | 0 | 0 | 0 | 0 | 0 | 4 | Moderate |
| Soldan et al. 2016 | 1 | 1 | 1 | 1 | 0 | 0 | 0 | 0 | 0 | 4 | Moderate |
| Taylor et al. 2017 | 1 | 1 | 1 | 1 | 0 | 0 | 0 | 0 | 0 | 4 | Moderate |
| Um et al. 2017 | 0 | 1 | 1 | 1 | 0 | 0 | 0 | 0 | 0 | 3 | Low |
| Van Harten et al. 2013 | 0 | 0 | 1 | 1 | 0 | 0 | 0 | 0 | 0 | 2 | Low |
| Visser et al. 2009 CN | 1 | 0 | 1 | 1 | 0 | 0 | 0 | 0 | 0 | 3 | Low |
| SCD | 0 | 0 | 1 | 1 | 0 | 0 | 0 | 0 | 0 | 2 | Low |
| Wolfsgruber et al. 2015 | 1 | 1 | 1 | 1 | 0 | 0 | 0 | 0 | 0 | 4 | Moderate |
| Zhao et al. 2017 | 1 | 0 | 1 | 1 | 0 | 0 | 0 | 0 | 0 | 3 | Low |

Hoy D, Brooks P, Woolf A, Blyth F, March L, Bain C, et al. Assessing risk of bias in prevalence studies: Modification of an existing tool and evidence of interrater agreement. J Clin Epidemiol. Elsevier Inc; 2012;65:934–9.
